# Molecular characterization of Newcastle disease virus vaccines in Nigeria

**DOI:** 10.14202/vetworld.2022.2816-2821

**Published:** 2022-12-10

**Authors:** Mohammed Usman Sajo, Lawal Sa’idu, Maman Moutari Souley, Olusegun Adesina Fagbohun

**Affiliations:** 1 Institute of Life and Earth Sciences, Pan African University, (PAULESI), Ibadan, Nigeria; 2 Department of Veterinary Microbiology, University of Ibadan, Ibadan, Nigeria; 3 Department of Veterinary Microbiology, University of Maiduguri, Maiduguri, Nigeria; 4 Veterinary Teaching Hospital, Ahmadu Bello University, Zaria, Nigeria; 5 Laboratoire Central de L’Elevage, B.P: 485, Niamey, Niger Republic

**Keywords:** Newcastle disease virus, Nigeria, polymerase chain reaction, vaccines

## Abstract

**Background and Aim:**

Newcastle disease (ND) caused by ND virus (NDV) is a serious impediment to effective poultry production in developing countries such as Nigeria. Despite employing vaccination and other control measures to curtail this disease, its severe forms still persist. This study aimed to confirm the virus strains in the NDV vaccine brands commonly used in Nigeria.

**Materials and Methods:**

We employed reverse-transcription polymerase chain reaction (RT-PCR), sequencing, and sequence analysis to characterize NDV strains in four NDV vaccines commonly used in Nigeria. Fragments of 300 bp from NDV fusion genes from the vaccines were amplified. Polymerase chain reaction products were sequenced and analyzed using multiple sequence alignment and phylogenetic analyses to characterize the vaccine viruses as pathotypes.

**Results:**

All the vaccines gave positive results, confirming the presence of NDV. Multiple sequence alignment and phylogenetic analyses revealed that two of the vaccines had the lentogenic pathotype, while the other two had the mesogenic or velogenic pathotype.

**Conclusion:**

This study provides information to facilitate strategies for regular control of the quality of vaccines in Nigeria.

## Introduction

The poultry industry in Nigeria provides cheap white meat and eggs as sources of protein and energy for the country’s rapidly growing population. However, an obstacle to the growth of this industry is diseases such as Newcastle disease (ND), which causes huge economic losses [1]. Newcastle disease is a highly contagious disease of poultry worldwide [2] and was first reported in Nigeria in 1953 [3]. Since then, outbreaks of the disease have been frequently reported in the country [4–6]. Clinical signs of NDV can be neurological, gastrointestinal, reproductive, or respiratory [7] and are frequently observed in naïve, unvaccinated, or poorly vaccinated birds. Newcastle disease is caused by ND virus (NDV) or avian paramyxovirus type 1 (APMV-1), a pleomorphic, enveloped, linear, single-stranded, non-segmented, and negative-sense genomic RNA virus of about 200–300 nm in diameter. This virus belongs to the family Paramyxoviridae, subfamily Avulavirinae, and genus *Orthoavulavirus* [8]. In terms of its organization, the NDV genome contains a 55-nucleotide (NT) leader at its 3′ end and a 114-NT trailer at its 5′ end, flanking genes encoding six structural proteins: Nucleocapsid (N), matrix protein, phosphoprotein, fusion protein (F), hemagglutinin-neuraminidase protein (HN), and large polymerase protein [9]. The F protein plays a pivotal role in the virulence and tropism of strains of the virus because of the amino acid composition of the fusion protein cleavage (Fc) site. The F protein is synthesized as an inactive precursor (F0), which is cleaved into two disulfide-linked polypeptides, F1 and F2, by host proteases at the Fcs. This cleavage results in exposure of the F peptide at the N terminus of the F1 subunit, which triggers the fusogenic activity of the virus [10]. Newcastle disease virus has been classified into three pathotypes, lentogenic, mesogenic, and velogenic, based on disease severity. Lentogenic NDV strains do not cause disease in adult birds and are regarded as avirulent. Such strains include LaSota and Hitchner B1 NDVs [11]. Meanwhile, mesogenic strains cause moderate infections, which are mainly respiratory, an example of which is the Anhinga strain [12]. Velogenic strains including ZJI and Herts/33 [13, 14] are responsible for highly virulent disease and have been reported worldwide [15]. Lentogenic strains of NDV possess a monobasic amino acid motif at the virus Fc, ^112^GR/K-Q-G-R↓L^117^, and are cleaved extracellularly by trypsin-like proteases present in the host’s respiratory and intestinal tract. In contrast, mesogenic and velogenic strains have a multibasic amino acid motif, ^112^R/G/KR-Q/K-K/R-R↓F^117^ and are cleaved intracellularly by ubiquitous furin-like proteases [16, 17]. This region is usually used to type NDVs into pathotypes molecularly [18, 19].

Biosecurity and vaccination are two approaches to ensure effective control of ND. Biosecurity involves various techniques, such as cleaning, disinfecting, preventing farm access by rodents and wild birds, and strict hygiene practices among visitors and farm staff [20]. In chickens vaccinated with a combination of live and inactivated vaccines, a strong level of immunity is exhibited compared with that in chickens receiving only live vaccines; meanwhile, genotype-matched vaccines provide better protective immunity than heterogeneous vaccine strains [21]. Live LaSota and B1 vaccines were first developed based on lentogenic NDV strains and are the vaccine strains that are most commonly used worldwide [22]. Despite various vaccination efforts against ND in Nigeria, the disease still persists.

This study aimed to confirm the pathotypes of vaccines commonly used to vaccinate poultry against this disease in Nigeria so as to provide information that will bolster strategies on frequent quality control of vaccines in the country. Consequently, we employed the Fc region of the F gene to characterize the commonly used NDV vaccines in Nigeria as pathotypes molecularly.

## Materials and Methods

### Ethical approval

The study was approved by the Ethics Committee of Laboratoire Central de L’Elevage.

### Study period and location

The study was conducted from December 2018 to March 2019 in Laboratoire Central de L’Elevage.

### NDV vaccine sampling

One vial of each of the following four commonly used brands of NDV vaccines in Nigeria was analyzed for molecular characterization: NDVac_NVRI13 (Nigeria), NDVac_LAPRO14 (Hungary), NDVac_RAN15 (India), and NDVac_I216 (Ghana).

### Reverse-transcription polymerase chain reaction (RT-PCR) and sequence analysis

#### Viral RNA extraction

Total RNA was extracted from the vaccines using QIAGEN RNeasy® Mini Kit (QIAgen, Hilden, Germany), in accordance with the manufacturer’s instructions. The extracted RNA was kept at −80°C until analysis.

#### One-step RT-PCR

One-step RT-PCR was performed to amplify a 300 bp fragment of the F gene using the following primers for the genotyping and pathotyping of APMV-1: NOH-forward: 5´-TACACCTCATCCCAGACAGG-3´ and NOH-reverse: 5´-AGTCGGAGGATGTTGGCAGC-3´ [23]. Using a final reaction volume of 25 μL, reaction mixtures contained 13.4 μL of RNase-free water, 5 μL of PCR buffer 5×, 0.5 μL of dNTPs, 0.5 μL of One-step RT-PCR Enzyme Mix, 0.1 μL of RNase inhibitor, 5 μL of RNA template, and 1 μL of each primer. A 96-well thermocycler (Applied Biosystems 2720) was used for the amplification, initially involving reverse-transcription at 50°C for 30 min. This was followed by initial denaturation at 94°C for 15 min; 35 cycles of denaturation at 94°C for 30 s, annealing at 55°C for 1 min, and elongation of the templates at 68°C for 1 min; and then final extension of the templates at 68°C for 7 min. Nuclease-free water without an RNA template was used as a negative control. The products were electrophoresed at 80 V for 40 min in 1.5% agarose gel in Tris-acetate ethylenediaminetetraacetic acid and PCR bands were visualized under an ultraviolet transilluminator (ENDURO™ GDS Labnet, Aplagen). The PCR products were purified using Wizard® SV Gel and PCR Clean-Up System (Promega, Madison, WI, USA) in accordance with the manufacturer’s instructions. Then, automated NT sequencing was carried out on an ABI 3130XL. Chromas 2.6.6 (Technelysium, South Brisbane, Australia) was used to view and edit NT sequences.

#### Sequence analysis

The four purified PCR products were sequenced and deposited in GenBank with accession numbers MN488597 to MN488600. These NT sequences were compared with other published NDV sequences already available in the GenBank database using the BLAST search tool from the National Center of Biotechnology Information’s website (http://www.ncbi.nlm.nih.gov/BLAST/). The multiple sequence alignment was performed with the Clustal W algorithm in the CLC Main Workbench (Qiagen, Valecia, CA, USA). Multiple sequence alignments of the NT sequences of the four vaccines from this study and the following six sequences retrieved from GenBank were performed: NDV LaSota (JF950510), NDV Hitchner-B1 (AF309418), NDV Komarov (KT445901), NDV Beaudette C (X04719), NDV VRD Ibadan/2/1973 (MH996952), and NDV Herts/33 (AY741404). The NDV vaccine virus F gene sequences were translated into amino acids using the ExPASy-Translate tool (https://web.expasy.org/translate/). Multiple sequence alignment of the deduced amino acid sequences was also carried out. A phylogenetic tree was constructed using the maximum likelihood method coupled with the Kimura two-parameter model with bootstrap analysis of 1000 replicates in MEGA, version 7.0 (https://www.megasoftware.net). The analysis involved four vaccine virus sequences and 10 sequences retrieved from GenBank, namely, NDV LaSota (JF950510), NDV Hitchner-B1 (AF309418), NDV Komarov (KT445901), NDV Beaudette C (X04719), NDV VRD Ibadan/2/1973 (MH996952), NDV Herts/33 (AY741404), NDV Roakin (AY289000), NDV Local chicken/Nigeria (MH996911), NDV Chicken/Nigeria (MH996993), and NDV KA697 (MN096603), along with NDV HN (DQ013889) as an outgroup. WebLogo 3.1 (http://weblogo.threeplusone.com/create.cgi) [24, 25] was also employed to give a richer and more precise description of the vaccine’s Fc. VaxiJen server (http://www.ddg-pharmfac.net/vaxijen/VaxiJen/VaxiJen.html) [26], an alignment-independent server for predicting protective antigens, was used to predict the antigenicity of an 85-amino-acid region of the F protein harboring the Fcs, corresponding to positions 67–151 in each virus strain.

## Results

### Polymerase chain reaction and sequence analysis

**Figure-1:**
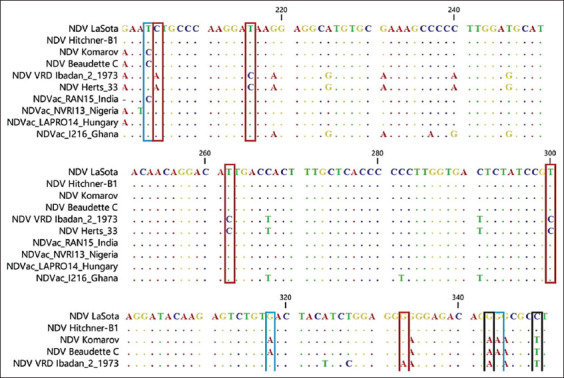
Multiple sequence alignment of the nucleotide sequences of partial F protein of Newcastle disease virus (NDV) vaccine virus from this study and NDV sequences retrieved from the GenBank. The NDV vaccine sequences here analyzed are NDV_RAN15, NDV_NVRI13, NDV_LAPRO14, and NDV_1213. These were compared with sequences of NDV LaSota, NDV Hitchner-B1, NDV Komarov, NDV Beaudette, C, NDV Herts/33 and NDV VRD Ibadan/2/1973 of known pathotypes retrieved from the GenBank. Key positions that can be used to distinguish the pathotypes are shown in boxes with red, blue and black depicting velogenic, mesogenic and lentogenic, respectively. The region encoding the Fc, nucleotide positions 330–351 (equivalent to amino acid positions 112–119) is demarcated with red dotted lines.

**Figure-2:**
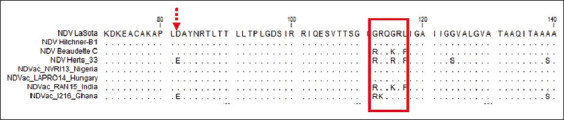
Multiple sequence alignment of the deduced amino acid sequences of fragments of F protein gene of Newcastle disease virus (NDV). The F proteins of NDV vaccine sequences here analyzed are NDV_RAN15, NDV_NVRI13, NDV_LAPRO14, and NDV_1213. Reference strains are: NDV LaSota, NDV Hitchner-B1, NDV Komarov, NDV Beaudette, C, NDV Herts/33 and NDV VRD Ibadan/2/1973 of known pathotypes retrieved from the GenBank. Dots indicate position where the F protein sequences are identical to that of the consensus sequence. The Fc amino acids 112–117 which determine NDV pathotypes are shown in the red box. Red dotted arrow indicates substitution D82E for the velogenic pathotype.

**Figure-3:**
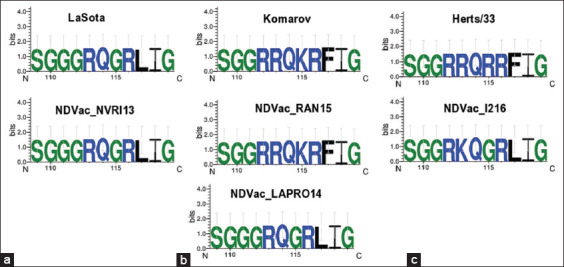
Sequence analysis of Fcs variants at the fusion protein cleavage site in Newcastle disease virus (NDV). Diversity of residues at each position of the Fcs were analyzed using WebLogo 3.1 (http://weblogo.threeplusone.com/create.cgi). (a) Lentogenic pathotypes. (b) Mesogenic pathotype (c) Velogenic pathotype.

**Figure-4:**
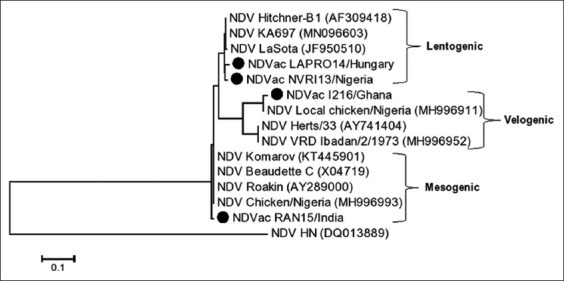
Phylogenetic analysis of Newcastle disease virus (NDV) based on F gene nucleotide sequences. Phylogenetic tree was constructed via multiple alignments of nucleotide sequence of F gene from Nigerian NDV and vaccine sequences. The tree was analyzed by maximum likelihood method with bootstrapping (1000). Bar, 0.1 nucleotide substitutions per site. Sequences from this study are depicted with black circles.

Fragments of 300 bp of the virus F gene were amplified for each of the four vaccine samples using RT-PCR. The results showed that all the samples were positive for NDV. Multiple sequence alignments of the four vaccines’ NT sequences from this study and six sequences retrieved from GenBank were performed. As shown in Figure-1, positions 343, 349, and 376 can be used to differentiate lentogenic strains from the other two pathotypes. Meanwhile, the NT substitutions C205A, T216C, T263C, T300C, G370A, A402G, A408G, T420C, and A438G distinguish the velogenic strain from the other pathotypes. Taking the findings together, positions 336–351 are important for characterizing the pathotypes. Multiple sequence alignment of the deduced amino acid sequences was also performed. Amino acid analysis at the Fc showed that NDV-NVR113 (Nigeria) and NDV-LAPRO14 (Hungary) had the motif ^112^G-R-Q-G-R↓L^117^, indicating that they are lentogenic strains. Meanwhile, NDV-RAN15 (India) had ^112^R-R-Q-K-R↓F^117^, revealing that it is a mesogenic strain. However, NDV_1216 had the motif ^112^R-K-Q-G-R↓L^117^, showing that it is a velogenic strain because of the two basic amino acids arginine and lysine (Figure-2). WebLogo analysis of the deduced amino acids further confirmed the multiple sequence alignment results (Figure-3). The phylogenetic tree showed that all the four vaccine study sequences were grouped together with representative pathotypes (Figure-4). VaxiJen server antigenicity prediction revealed that the vaccines have higher antigenicity than their respective standard pathotype strains, except NDV_Vac1216.

## Discussion

**Figure-5:**
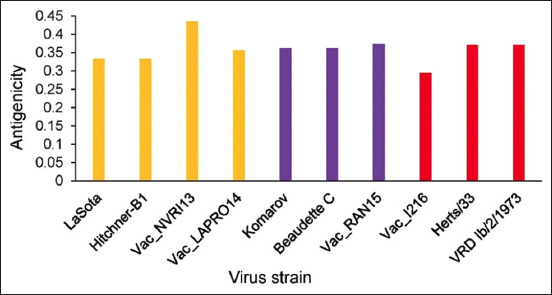
Fusion protein fragment antigenicity prediction using VaxiJen server. Antigenicity of an 85 amino acid residue of the fusion protein containing the Fcs, positions 67–151 of the vaccine viruses (Vac_NVRI13, Vac_LAPRO14, Vac_RAN15, and Vac_1216) and the reference strains (LaSota, Hitchner-B1, Komarov, Beaudette C, Herts/33, and VRD Ib/2/1973).

Newcastle disease has been enzootic in Nigeria for almost 70 years since the first reported case in 1953. Despite vaccination efforts, small disease outbreaks still occur in the country. There is thus a need to confirm the virus pathotypes in commonly used vaccines, for vaccine quality control. This can be achieved by RT-PCR, multiple sequence alignment of the NT or deduced amino acid sequences, and phylogenetic reconstruction. Therefore, in this study, we used RT-PCR and sequencing analyses to confirm the pathotypes of NDV vaccines commonly used in Nigeria. All the vaccines tested were confirmed to be positive for NDV. Multiple sequence alignment of the NT s of a 300 bp fragment of F proteins of the standard representatives of the virus pathotypes and of the four vaccines revealed that positions 205, 216, 222, 300, 373, 390, 402, 408, 420, and 438, besides the Fc site 330–351, can be used to differentiate the pathotypes (Figure-1). Multiple sequence alignment of the deduced amino acids revealed the Fc site (Figure-2), which showed Vac-NVR13 and Vac_LAPRO14 as being of the lentogenic pathotype, Vac_RAN15 as mesogenic, and Vac_1216 as velogenic. Besides the Fc amino acid changes, amino acid substitution D82E may also be responsible for the velogenic status of Vac_1216 (Figure-2). WebLogo analysis of amino acid positions 109–119 encompassing the Fc bolstered the aforementioned classification of the vaccine’s viruses (Figure-4). The unique lentogenic motif ^112^G-R-Q-G-R↓L^117^ was present in Vac_NVRI13 and Vac_LAPRO14 and the mesogenic motif ^112^R-R-Q-K-R↓F^117^ was present in Vac_RAN15. However, the motif ^112^R-K-Q-G-R↓L^117^ was observed in Vac_1216, instead of motif ^112^R-R-Q-G-R↓F^117^ commonly present in velogenic pathotypes. Its velogenicity may be due to R113K because lysine (K) is a basic amino acid like arginine (R), which may affect the motif’s properties. This may imply that phenylalanine (F) at position 117 present in mesogenic and velogenic pathotypes may not be solely responsible for the pathotypes’ virulence. Furthermore, according to the World Organization for Animal Health, the presence of at least three basic amino acids at the Fc is indicative of a virulent form of NDV [27, 28]. From this study’s results, the mesogenic pathotype vaccine appears to be closer to the velogenic pathotype than to the lentogenic pathotype. Phylogenetic analysis of the four vaccine viruses, three representatives of the pathotypes and sequences retrieved from GenBank, further confirmed the pathotypes, with Vac_NVRI13 and Vac_LAPRO14 clustering with the lentogenic pathotypes, Vac_RAN15 clustering with the mesogenic pathotypes, and Vac_1216 clustering with the velogenic pathotypes. Evaluation of the antigenicity of the viruses at positions 67–151 of the F protein harboring Fc using VaxiJen server revealed that the vaccine viruses were more antigenic than the representative of the pathotypes, with the exception of Vac_1216 showing the lowest antigenicity (Figure-5). Although a fragment of the F protein was used for the analysis, a better result could be achieved using the full-length F protein. Furthermore, the results are based on computational prediction, so more reliable findings could be obtained through experimental determination of the antigenicity.

## Conclusion

This study revealed the presence and pathotypes of NDV in four of the major vaccines used in Nigeria to vaccinate poultry against NDV. Two of the vaccines contained lentogenic NDV pathotypes, whereas the other two contained mesogenic and velogenic pathotypes. This information is important for eradicating ND through the implementation of effective control policies.

## Authors’ Contributions

MUS: Designed the study, collected samples, analyzed data, and wrote the manuscript. LS and OAF: Supervised the study, analyzed data, and edited the manuscript. MMS: Provided critical advice and skills on the laboratory activities. All authors have read and approved the final manuscript.
